# Chemical Composition and Assessment of the Anti-Inflammatory, Antioxidant, Cytotoxic and Skin Enzyme Inhibitory Activities of *Citrus sinensis* (L.) Osbeck Essential Oil and Its Major Compound Limonene

**DOI:** 10.3390/ph17121652

**Published:** 2024-12-08

**Authors:** Naoufal El Hachlafi, Amine Elbouzidi, Amine Batbat, Mohamed Taibi, Mohamed Jeddi, Mohamed Addi, Hanae Naceiri Mrabti, Kawtar Fikri-Benbrahim

**Affiliations:** 1 Laboratory of Microbial Biotechnology and Bioactive Molecules, Faculty of Sciences and Technologies, Sidi Mohamed Ben Abdellah University, Imouzzer Road, Fez 30000, Morocco; 2 Laboratoire d’Amélioration des Productions Agricoles, Biotechnologie et Environnement (LAPABE), Faculté des Sciences, Morocco des Sciences, Université Mohammed Premier, Oujda 60000, Morocco; 3 Laboratory of Applied Organic Chemistry, Faculty of Sciences and Techniques, Sidi Mohamed Ben Abdellah University, Route d’Imouzzer, Fez 30000, Morocco; 4 Centre de l’Oriental des Sciences et Technologies de l’Eau et de l’Environnement (COSTEE), Université Mohammed Premier, Oujda 60000, Morocco; 5 High Institute of Nursing Professions and Health Techniques Casablanca, Casablanca 20250, Morocco

**Keywords:** *Citrus sinensis*, limonene, antioxidant activity, anti-inflammatory, cytotoxicity, dermatoprotection, cosmeceuticals, oxidative stress, skin health

## Abstract

**Background/Objectives:** Essential oils (EOs) from *Citrus* species have attracted attention for their diverse properties, including anti-inflammatory, antioxidant and cytotoxic effects, which address critical health challenges such as chronic diseases and skin disorders. *Citrus sinensis* (L.) Osbeck, which is a widely cultivated citrus fruit, is attracting increasing attention in the field of medicinal research due to its richness of limonene (comprising approximately 85–90% of the oil). This study investigates the chemical profile of CS-EO and biological activities of CS-EO and limonene. **Methods and Results:** This study used gas chromatography–mass spectrometry (GC-MS), confirming limonene as the predominant compound (70.15%) along with other minor constituents, including thujene (10.52%), myrcene (5.54%) and α-pinene (2.81%). The biological activities of CS-EO and limonene were examined, specifically focusing on their antioxidant, anti-inflammatory, cytotoxicity and dermatoprotective effects. Antioxidant potential was evaluated using DPPH, FRAP and beta-carotene assays, with CS-EO and limonene exhibiting comparable efficacy. Anti-inflammatory properties were assessed via inhibition assays of prostaglandin E2 (PGE2) and nitric oxide (NO) production, showing significant reductions in LPS-stimulated macrophages treated by CS-EO or limonene. Cytotoxicity testing on various cell lines indicated selective activity of the tested compounds, with low toxicity observed on human skin fibroblasts. Limonene and CS-EO were highly effective on HepG2 cellules, with IC_50_ values of 0.55 ± 0.01 µg/mL and 15.97 ± 1.20 µg/mL, respectively. Dermatoprotective effects were further confirmed using enzymes, where CS-EO and limonene showed remarkable inhibitory potential against elastase (IC_50_ of 65.72 ± 1.92 and 86.07 ± 1.53 µg/mL, respectively) and tyrosinase (IC_50_ of 102 ± 2.16 and 78.34 ± 1.15 µg/mL, respectively) enzymes compared to quercetin used as a standard (IC_50_ of 111.03 ± 0.1 and 124.22 ± 0.07 µg/mL, respectively). **Conclusions:** The findings of this study suggest the potential for the development of new therapeutic approaches based on CS-EO, which could be applicable in the pharmaceutical, cosmetic and nutraceutical fields and have protective benefits for skin health.

## 1. Introduction

Natural substances, especially those originating from medical herbs, have constituted an almost inexhaustible reservoir of bioactive substances during the course of human history [1,2]. Among such natural resources, those of the genus *Citrus*, and particularly *Citrus sinensis* (sweet orange), are notable for their abundance of pharmacologically active constituents. The essential oil (EO) derived by extraction from *C. sinensis* is not only a valuable secondary metabolite in the *Citrus* industry but also a rich source of therapeutic compounds [3,4,5]. Numerous research studies have been conducted on the essential oil of *C. sinensis* with the aim of elucidating its detailed chemical composition. These studies have consistently demonstrated the prevalence of cyclic monoterpenes, with limonene representing the majority of the total content, accounting for 90% of the total [6]. Furthermore, GC-MS analyses have identified several significant minority compounds, including myrcene, linalool and α-pinene. This comprehensive characterization of the chemical composition is of critical importance in the biomedical field as it has facilitated the establishment of correlation between the content of these bioactive compounds and the biological properties observed [3,7]. The prevalence of limonene, in conjunction with the potential synergistic effects of minor compounds, explains the mounting interest in this essential oil in therapeutic research [8,9]. A substantial number of in vitro and in vivo investigations have substantiated the encouraging anticancer attributes of *C. sinensis* EO and limonene in the context of cancer research. These compounds exert their antitumor effects via a number of different mechanisms, including the induction of apoptosis in cancer cells, the inhibition of cell proliferation, the modulation of the cell cycle and the regulation of crucial signaling pathways involved in carcinogenesis [7,10,11]. In particular, limonene has demonstrated promising results in a number of cancer models, including those pertaining to breast, liver and colon cancers [12,13,14,15]. The EO of *C. sinensis* is notable for its substantial anti-inflammatory effects. In this context, *C sinensis* EO has been demonstrated to possess significant anti-inflammatory properties, which are achieved by modulating the production of pro-inflammatory mediators such as TNF- α, IL-1β and IL-6. The NF- κB and MAPk signaling pathways are pivotal in the inflammatory response [16,17], and while some EOs have been shown to affect the expression of anti-inflammatory genetic pathways [18,19], there is still a paucity of comprehensive studies that elucidate their bioactivity and mechanism of action in relevant biological systems. In addition, oxidative stress, defined as an imbalance between the production of reactive oxygen species (ROS) and the antioxidant capacity of cells, is a common pathological mechanism observed in numerous diseases [20]. *C. sinensis* EO has been demonstrated to possess notable antioxidant ability through a number of mechanisms, including the direct neutralization of free radicals [3,21]. In this context, beta-carotene, a precursor of vitamin A, is a potent antioxidant renowned for its capacity to neutralize free radicals, thereby reducing oxidative stress and protecting cells against damage linked to chronic diseases [22]. Its other effects include the enhancement of endogenous antioxidant defenses, such as superoxide dismutase (SOD) and catalase (CAT), and the modulation of gene expression involved in the response to oxidative stress [5,23]. Nevertheless, despite the extensive literature publications on the antioxidant potential of EO compounds, further research is necessary to elucidate their precise mechanisms of action and confirm their efficacy in complex biological systems. In the field of dermatoprotection, *C. sinensis* exhibits particularly noteworthy characteristics. The capacity of this EO to safeguard the skin against damage caused by UV radiation, stimulate collagen production and facilitate tissue regeneration has been substantiated by empirical evidence [9,24]. Furthermore, *C. sinensis* members are widely acknowledged for their efficacy as natural repellents against pests and insects, rendering them advantageous in agricultural and domestic applications [25,26].

Despite the well-documented therapeutic potential of EOs, in particular their biological and antioxidant properties, there is a paucity of studies that specifically focus on the bioactivities of CS-EO and its main compound (limonene). This study aims to investigate the chemical composition of *Citrus sinensis* essential oil (CS-EO) and to assess, for the first time, its pharmacological activities along with those of its primary component, limonene. Specifically, the research evaluates its anti-inflammatory effects in RAW 264.7 macrophages, antioxidant capacity, cytotoxic potential against human cancer cell lines and enzyme inhibition activities. Together, these findings are expected to provide a comprehensive understanding of the therapeutic potential of CS-EO and limonene across diverse biomedical applications. In light of the mounting interest in natural products for therapeutic applications and the rich composition of CS-EO, and by focusing on its major compound, limonene, this research aims to provide a deeper understanding of its bioactive potential and to support its application in the pharmaceutical and cosmetic industries.

## 2. Results and Discussion 

### 2.1. GC-MS Analysis of Volatile Compounds 

**Figure 1:**
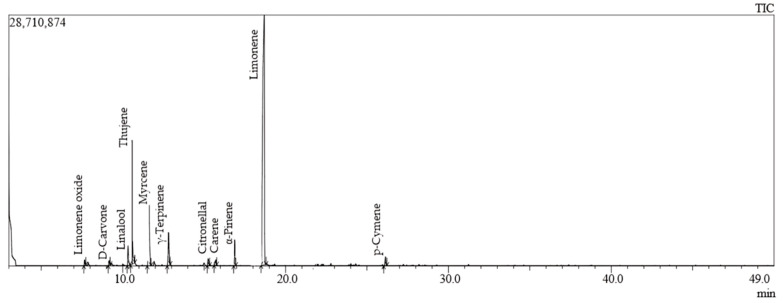
Chromatogram of GC-MS analysis of CS-EO.

CS-EO, as revealed by GC-MS analysis (Table 1, Figure 1), is constituted primarily by hydrocarbon monoterpenes, which account for 92.63% of the total content. The dominant constituent is D-limonene, with a notable proportion of 70.15%, followed by thujene (10.52%), myrcene (5.54%) and α-pinene (2.81%). The presence of oxygenated compounds is relatively minor (4.37%). Limonene has been identified as the principal compound in CS-EO, and this high prevalence serves to underscore its pivotal role in the biological activities observed in our study, including antioxidant, anti-inflammatory and cytotoxic properties.

CS-EO have been the subject of extensive scientific investigations. A number of analytical studies have been conducted on the chemical composition of this EO, yielding comparable results. In general, these oils are characterized by a particularly high concentration of limonene. Accordingly, an analysis of samples of *C. sinensis* collected in the Sindhuli district of Nepal detected that their composition was dominated by limonene, comprising more than 90% of the total oil content [27]. Similarly, a study conducted on oranges (*C. sinensis*) procured from the local market in Khartoum, Sudan, demonstrated that D-limonene constituted 95.39% of the EO composition [28]. More recently, research conducted in Argentina on CS-EO obtained by cold pressing followed by steam distillation corroborated the preponderance of limonene, which constituted between 89.8% and 90.04% of the total composition [29]. In addition, a comparable investigation in Turkey likewise demonstrated that limonene represented the predominant constituent, corresponding to 96.52–96.61% of the EO derived from *C. sinensis* [4]. Furthermore, an analysis of the chemical composition of EO, conducted by Hamdan and his colleagues, indicated that limonene (43.2%), β-pinene (44.5%) and sabinene (55.9%) constituted the primary constituents of the oil [30]. In a comparative study, two extraction methods were employed to obtain *C. sinensis* EO: hydrodistillation and solvent-free microwave extraction. The GC-MS analysis identified a similar profile. Limonene was reported as the predominant compound, representing 98.23% of the hydrodistilled oil and 98.41% of the solvent-free microwave-extracted oil [31]. In consideration of the aforementioned factors, it can be posited that the discrepancies in CS-EO composition may be attributed to a multitude of variables, including geographical provenance, climatic fluctuations and the circumstances surrounding harvesting and processing. Also a variety of ecological factors, environmental variance and fertilization can all exert an influence on the chemical composition of EOs [32,33]. It is, in fact, possible to hypothesize that the geographical coordinates of the culture site, including both latitude and altitude, may exert a modifying effect upon the temperature and exposure to solar radiation, all of which have the potential to affect the composition of the EO. This proposition is supported by the findings of previous studies [1,34,35,36]. 

### 2.2. Anti-Inflammatory Activity

**Figure 2:**
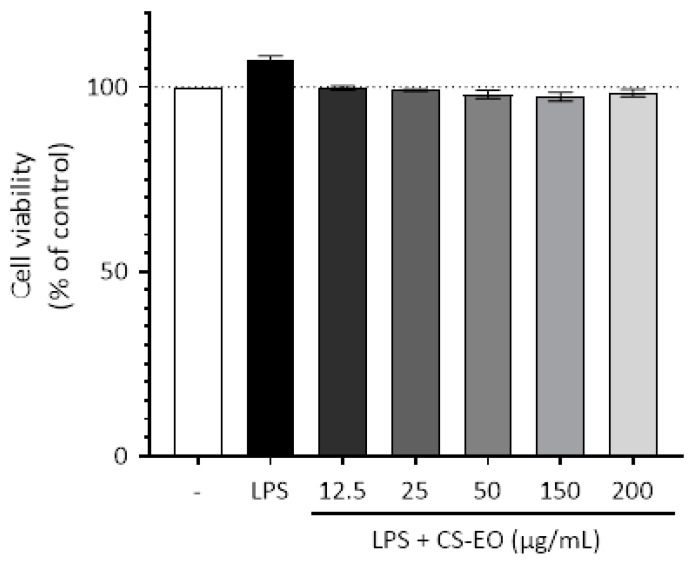
Effect of CS-EO on cell viability. RAW 264.7 cells were stimulated with LPS (1 μg/mL) and incubated in the presence or absence of increasing concentrations (25–200 µg/mL) of CS-EO for 24 h.

Assessing the survival of RAW 264.7 cells stimulated by LPS constitutes a pivotal endeavor in comprehending the influence exerted by CS-EO on the inflammatory response. As delineated in Figure 2, a conspicuous augmentation in the percentage of RAW 264.7 cells is evident subsequent to exposure to 1 µg/mL of LPS. Conversely, an abundance of RAW 264.7 cells remains unaltered with escalating concentrations of CS-EO, save for the fourth concentration (150 µg/mL), which induced a marginal decline in cellular viability to approximately 99%. Notably, concentrations up to 200 µg/mL elicit no discernible reduction in cellular viability, thus insinuating the non-cytotoxic nature of CS-EO on RAW 264.7 cells.

**Figure 3:**
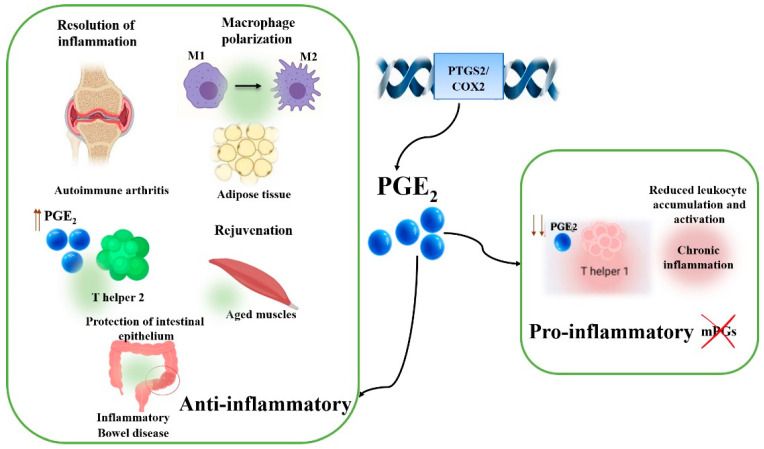
PGE_2_ as a modulator of immune dynamics in inflammation and resolution.

The fundamental aim of this study is to assess the inherent anti-inflammatory properties of CS-EO by orchestrating the production of key inflammatory mediators, namely, NO and PGE2. The crucial importance of NO and PGE2 in the context of inflammation is undeniable. NO acts as a central cellular messenger, regulating a plethora of biological processes including the inflammatory response [37]. This compound is generated by various immune cells in response to inflammatory stimuli and can exert either pro- or anti-inflammatory effects, depending on the pathophysiological context. In parallel, PGE2, as a pro-inflammatory prostaglandin, is abundantly produced during inflammatory episodes, actively participating in vasodilation, increased vascular permeability and nociceptor sensitization, all fundamental processes in the mediation of pain and inflammation [38]. Figure 3 illustrates PGE_2_’s dual actions in inflammation, emphasizing its role in acute inflammation as a promoter and in chronic inflammation as a modulator. The spatial and temporal dynamics of PGE_2_ are also pivotal for transitioning from inflammation initiation to resolution, highlighting its therapeutic potential in inflammatory and autoimmune diseases.

**Figure 4:**
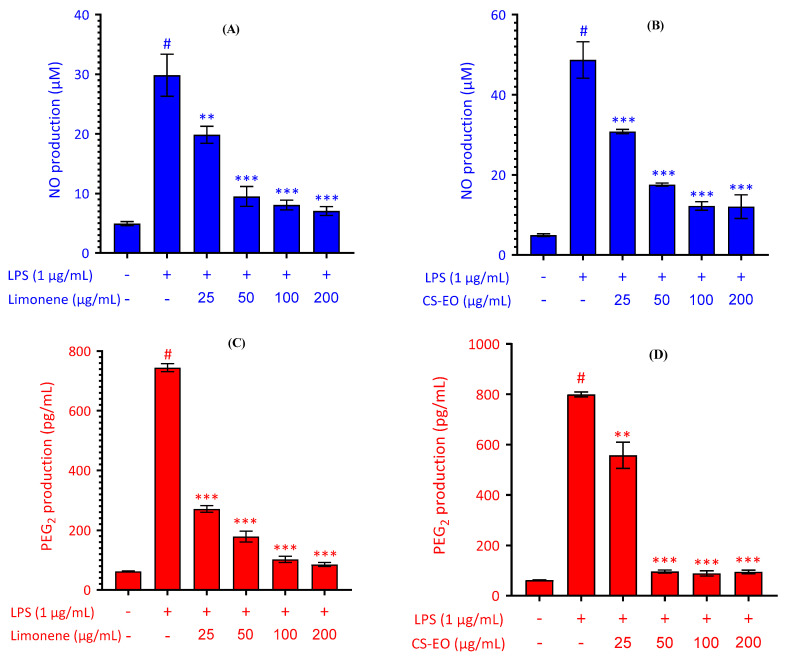
Effect of CS-EO and limonene on LPS-induced nitric oxide (NO) and prostaglandin E2 (PEG2) production. (**A**,**B**) RAW 264.7 cells were stimulated with LPS (1 μg/mL) and incubated in the presence or absence of increasing concentrations (25–200 µg/mL) of CS-EO and limonene (25–200 µg/mL) for 24 h. The nitrite concentration in the culture media was determined by the Griess reagent assay. (**C**,**D**) PGE_2_ levels in the culture media were measured by a commercially available assay kit. Data are reported as mean ± SD of three independent experiments. # *p* < 0.001 indicates a significant difference between the control and LPS-only treated groups. ** *p* < 0.01 and *** *p* < 0.001 show significant differences between the LPS-alone and CS-EO or limonene treatment groups.

By selectively intervening in NO and PGE2 production, it becomes possible to effectively modulate the inflammatory response, opening up promising new prospects for the development of innovative therapeutic strategies to counter inflammation. The data presented in Figure 4A,B detail the concentrations recorded under specific experimental conditions. Regarding Figure 4A, the control group shows a baseline NO production of 4.933 ± 0.3512 µM, while the addition of LPS results in a significant increase to 48.70 ± 4.544 µM. The simultaneous introduction of CS-EO, at concentrations ranging from 25 to 200 µg/mL with LPS at a dose of 1 µg/mL, demonstrates a dose-dependent decrease in NO production, with values ranging from 30.81 ± 0.545 to 12.07 ± 2.95 µM. Meanwhile, in Figure 4B, the control group reveals a PGE2 production of 62.03 ± 1.704 pg/mL, while LPS administration alone causes a significant increase to 799.70 ± 9.95 pg/mL. Incorporation of CS-EO at different concentrations induces a dose-dependent reduction in PGE2 production, with values ranging from 95.09 ± 6.96 to 111.60 ± 4.63 pg/mL.

These results suggest that CS-EO possesses anti-inflammatory activity, illustrating a dose-dependent efficacy in attenuating LPS-induced NO and PGE2 production. These findings underline the beneficial potential of CS-EO in modulating inflammatory processes. Analyses reveal a dose-dependent decrease in NO and PGE2 production in the presence of CS-EO, illustrating its ability to modulate inflammatory responses. This remarkable activity is probably attributed to bioactive compounds such as limonene, the main component of this essential oil. Several studies have demonstrated the efficacy of limonene as an anti-inflammatory agent [39,40]. To confirm these investigations, the evaluation of limonene was conducted following the same process as that used for assessing the studied essential oil. The results of Figure 4C,D depict the concentrations measured under specific experimental conditions. Figure 4C shows that the control group exhibits a basal production of NO at 4.933 ± 0.3512 µM, while the introduction of LPS leads to a substantial increase to 29.840 ± 3.533 µM. The simultaneous addition of limonene, at concentrations ranging from 25 to 200 µg/mL with LPS at a dose of 1 µg/mL, demonstrates a dose-dependent reduction in NO production, ranging from 19.850 ± 1.421 to 7.047 ± 0.743 µM. Similarly, in Figure 4D, the control group shows a production of PGE2 at 62.03 ± 1.704 pg/mL, while LPS alone significantly amplifies this production to 744.4 ± 13.410 pg/mL. The addition of limonene at varying concentrations induces a dose-dependent decrease in PGE2 production, ranging from 271.00 ± 11.18 to 85.50 ± 6.751 pg/mL. These results confirm the efficacy of limonene as an anti-inflammatory agent. They also highlight that the studied EO possesses notable anti-inflammatory activity due to its major compound, limonene. It is possible that other components of the studied essential oil may contribute to this activity.

Several studies have confirmed the anti-inflammatory efficacy of this particular essential oil. For example, Matuka et al. (2020) conducted a study highlighting the remarkable in vivo anti-inflammatory activity of CS-EO grown in South Africa [41].

All these findings suggest that CS-EO can be a promising biological alternative in the field of anti-inflammation. Its demonstrated effectiveness in modulating inflammatory responses and its safety towards the studied cells reinforce its therapeutic potential. These discoveries open new avenues in the search for natural and safe anti-inflammatory treatments.

### 2.3. Anticancer Activity

Evaluation of the anticancer efficacy of CS-EO on three distinct cell lines—MCF-7 (MCF-7 is considered a ‘triple positive’ breast cancer model because it expresses the hormone receptors estrogen (ER+) and progestins (PR+), as well as the HER2 receptor), HepG2 (a model of hepatocellular carcinoma) and HCT-15 (representing colorectal cancer)—as well as mononuclear blood cells (PBMC) gave promising results.

**Figure 5:**
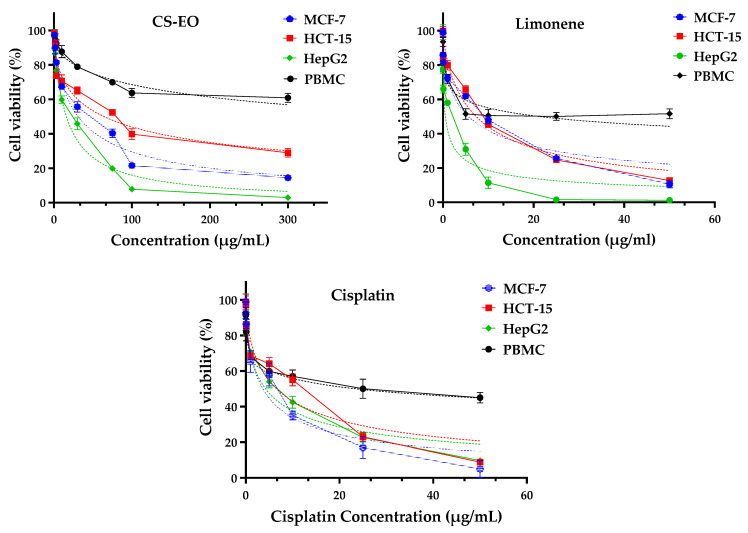
Cell viability of MCF-7, HCT-15, HepG2 and PBMC cells after 72 h of treatment with CS-EO, its respective major compound limonene and cisplatin (positive control) using MTT test.

The obtained results indicate significant anticancer activity of CS-EO against these specific cancer cell lines, as shown by IC_50_ values ranging from 15.97 ± 1.20 µg/mL against the HepG2 cell line to 64.11 ± 5.45 µg/mL against HCT-15 (Figure 5; Table 2). Of particular note was the selectivity observed against cancer cells compared to healthy PBMC (IC_50_ = 546.40 ± 6.72 µg/mL). Intriguingly, CS-EO showed higher selectivity indices (17.64 ± 0.05, 8.51 ± 0.20 and 34.17 ± 0.21 for MCF-7, HCT-15 and HepG2, respectively) than cisplatin, a chemotherapy drug widely used in the treatment of various types of cancer (6.46 ± 0.20, 3.57 ± 0.29, 5.53 ± 0.21) (Table 1). These selectivity indices underline the ability of this EO to selectively target cancer cells while minimizing the impact on healthy cells. These results suggest that CS-EO has significant anticancer activity, potentially rivalling or surpassing that of positive cisplatin treatment, with significant selectivity towards cancer cells.

In general, the anticancer activity of EOs is generally attributed to their majority compounds, which generally possess bioactive molecules. In this particular case, the majority compound in CS-EO is limonene (76.15%). To confirm this hypothesis, similar analyses were carried out to assess the anticancer activity of this compound. The results of the evaluation of the anticancer activity of limonene revealed notable performances, surpassing those of the EO, with respective IC_50_ values of 0.55 ± 0.01 against HepG2 and 7.32 ± 0.86 µg/mL against HCT-15. However, less selectivity was observed against healthy PMBC cells (IC_50_ = 21.35). Intriguingly, limonene showed higher selectivity indices (3.79 ± 0.19, 2.92 ± 0.34 and 38.82 ± 0.10 and 34.17 ± 0.21 µg/mL for MCF-7, HCT-15 and HepG2 and PBMC, respectively). 

These selectivity indices highlight the ability of limonene to selectively target cancer cells in the HepG2 line compared with the other lines tested.

These results corroborate the conclusions of Ghareeb et al. [42], who demonstrated the efficacy of the essential oil of Egyptian *C. sinensis* against various cancer cell lines. Similarly, Saengha et al. [43] also demonstrated the efficacy of this essential oil against various cell lines. This convergence of studies reinforces the credibility of the conclusions regarding the anticancer activity of CS-EO, opening up promising prospects for its potential use as a therapeutic agent against different types of cancer [42,43].

Furthermore, several other studies have also demonstrated the efficacy of limonene against various cell lines, underscoring the effectiveness of this bioactive molecule against diverse cellular lineages. These results corroborate the findings observed in our study [40,43,44].

All these results suggest that CS-EO could represent a promising natural alternative in the search for new products with anticancer activity. With its main compound, limonene, showing significant effects against different cancer cell lines, this EO has interesting potential for the development of natural anticancer therapies. This finding underlines the importance of continuing studies and research to further explore the mechanisms of action and efficacy of CS-EO in the treatment of cancer. It also paves the way for in-depth investigations into other compounds present in EOs and their potential applications in the fight against cancer.

### 2.4. Antioxidant Activity 

Natural products derived from medicinal plants have attracted a great deal of interest because of their powerful antioxidant properties. They act by neutralizing free radicals, i.e., unstable molecules that can induce oxidative damage to cells which contributes to health problems such as inflammation, aging and chronic diseases [45,46]. As demand for natural antioxidants increases due to consumer preference for alternatives to synthetic additives in food, cosmetics and pharmaceuticals, EOs and their monoterpene constituents have emerged as key agents in this field [47]. These plant-derived substances possess substantial antioxidant activity via a number of mechanisms, including the scavenging of free radicals, inhibition of lipid peroxidation and chelation of metal ions, thereby protecting cellular integrity and promoting overall health [48,49,50]. The rich diversity of phytochemical compounds occurring in essential oils not only reinforces their effectiveness as natural preservatives and health-promoting agents but also underscores their promising uses in a variety of industries, including nutraceuticals and skincare, where they can be safe and beneficial alternatives to synthetic antioxidants.

In this study, the IC_50_ values of CS-EO, limonene and BHT offer important insights into their efficacy in neutralizing free radicals and protecting against oxidative stress across three different assays, including DPPH, FRAP and β-carotene (Table 3).

Indeed, CS-EO exhibits the highest anti-DPPH ability with an IC_50_ of 82.54 ± 2.11 µg/mL, indicating its strong ability to scavenge free radicals compared with limonene (IC_50_ = 93.16 ± 1.56 µg/mL). This effect is competitive with that of BHT (IC_50_ = 77.06 ± 0.03 µg/mL), which positively indicates its effectiveness. Concerning the β-carotene test, CS-EO excels, with an IC_50_ of 67.48 ± 2.05 µg/mL, which exceeds that of the standard antioxidant BHT (75.24 ± 0.09 µg/mL), indicating its effectiveness in preventing the oxidation of β-carotene, which is crucial for skin protection. Limonene, with an IC_50_ of 105.09 ± 1.26 µg/mL, shows moderate protective efficacy in this assay. There are several possible explanations for the higher antioxidant potential of CS-EO compared to its major component limonene. In fact, one important point to note is that a plant’s EO comprises a number of bioactive compounds, some of which may act synergistically to boost overall activity. 

On the other hand, in the FRAP assay, limonene (IC_50_ = 81.10 ± 2.21 µg/mL) appears to be more effective than CS-EO (IC_50_ = 117.35 ± 3.67 µg/mL), providing insight into its potent antioxidant capacity when it comes to reducing ferric ions. This suggests that this molecule, as the main active component of CS-EO, plays a significant role in its overall antioxidant potency. However, the presence of additional components in the CS-EO could lead to interactions that slightly diminish the total antioxidant power compared to pure limonene. Several investigations have demonstrated the antioxidant capacities of CS-EO through multiple in vitro systems [51,52,53,54]. 

Limonene has also been identified as a potential antioxidant agent, which indicates the protective ability of normal lymphocytes against oxidative stress induced by H_2_O_2_ [55]. Indeed, limonene controls H_2_O_2_ levels, driving an increase in the activity of antioxidant enzymes such as catalase and peroxidase [55]. This event results in a biphasic effect on cell proliferation, where lower H_2_O_2_ levels correlate with enhanced cell growth. Moreover, another study reported that limonene counteracts the production of ROS induced by amyloid-beta (Aβ) oligomers [56]. This action not only promoted cell death in primary cortical neurons but also suppressed the hyperactivity of potassium channels that contribute to neuronal damage. Limonene has also been reported to exert interesting antioxidant potential through DPPH, ABTS, FRAP and other in vitro tests [57,58,59].

These findings provide evidence on the role of limonene as a bioactive agent in the prevention of diseases linked to oxidative damage, such as cancer inflammatory disorders, including neurodegenerative ones.

### 2.5. Dermatoprotective Potential 

In the current exploration, CS-EO has shown remarkable dermatoprotective effects, particularly in its ability to inhibit tyrosinase and elastase, which are the main enzymes involved in skin pigmentation and aging (Table 4). The IC_50_ values for CS-EO tyrosinase and elastase inhibition are 65.72 ± 1.92 and 102 ± 2.16 µg/mL. In comparison, limonene, a major component of CS-EO, exhibits IC_50_ values of 86.07 ± 1.53 µg/mL and 78.34 ± 1.15 µg/mL for the respective tyrosinase and elastase inhibitions. While it still displays inhibitory action, it suggests that other bioactive compounds identified in CS-EO contribute to its enhanced efficacy. On the other hand, limonene is more effective in inhibiting elastase, requiring only 78.34 ± 1.15 µg/mL to inhibit 50% of the enzyme activity. This effective inhibitory effect demonstrated that limonene may serve as a valuable agent for formulations targeting skin aging.

In contrast, quercetin, which is often recognized for its antioxidant and enzyme-inhibitory potency, possesses the least activity in terms of enzyme inhibition among the tested substances in the context of this specific investigation. It presents IC_50_ values of 111.03 ± 0.1 µg/mL and 124.22 ± 0.07 µg/mL for tyrosinase and elastase inhibition, respectively. 

To the best of our knowledge, no published works have studied the dermatoprotective activity of CS-EO through the inhibition of the tested enzyme, which makes our findings original in this field. However, some reports have assessed the ability of this oil to improve wound healing [60,61]. Regarding limonene, Kulig and his colleagues [62] reported a significant effect on inhibiting tyrosinase activity. Another study revealed the effectiveness of this bioactive component in preventing sunburn on mouse skin subjected to UV radiation. Following four days of oral administration of this compound at various doses, decreases in cell proliferation and sunburn were observed [63]. Recently, El Omari et al. [57] showed that limonene possesses effective action on both tyrosinase (IC_50_= 74.24 ± 2.06 µg/mL) and elastase (IC_50_= 91.25 ± 3.06 µg/mL) enzymes. 

Skin protection involves a process whereby certain compounds protect the skin against the effects of environmental stressors, aging and biochemical processes that may cause harm or degradation to the structure and function of the skin [64]. EOs and their monoterpenes are examples of dermatoprotective agents, mainly because they are capable of inhibiting enzymes such as tyrosinase and elastase, which are responsible for skin pigmentation and elasticity, respectively [65,66,67]. Tyrosinase is a key enzyme in the synthesis of melanin, and its excessive activity often leads to hyperpigmentation and uneven skin tone [66]. Monoterpenes such as limonene and linalool found in EOs act as tyrosinase inhibitors by interfering with the enzyme’s active site, thereby preventing the overproduction of melanin and promoting a more even skin tone [57]. Furthermore, elastase, the enzyme which breaks down elastin fibers, leads to sagging skin and the formation of wrinkles when it is too active, due in particular to aging or exposure to UV rays [68]. EOs have been found to inhibit elastase, preserving the skin’s natural elasticity and firmness by preventing the breakdown of elastin [69,70]. In addition to helping to reduce the signs of aging and pigmentation, this dual action on tyrosinase and elastase also highlights the considerable dermatoprotective potential of EOs in skincare, where they offer a natural, antioxidant-rich and multifunctional approach to managing the skin’s health and appearance.

**Figure 6:**
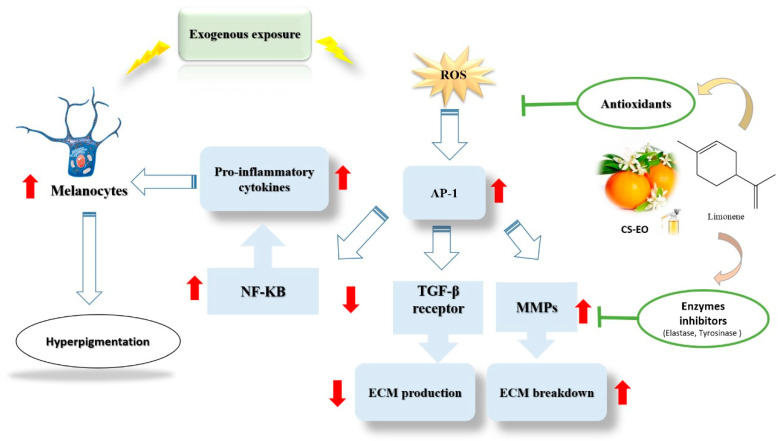
Schematic illustration of the possible mechanisms of action of CS-EO and limonene.

Taken together, our findings prove that both CS-EO and its main compound, limonene, possess significant potential as active ingredients in cosmeceutical products aimed at improving skin pigmentation and combating signs of aging. Their differing mechanisms of action and efficacy (Figure 6) suggest that they could be used synergistically in formulations to improve overall skin health. This warrants further investigation into their combined effects and underlying mechanisms, which could lead to the development of more effective skincare products targeting pigmentation and skin aging concerns. Although the present work is primarily concerned with the overall biological activity of CS-EO and its principal compound, limonene, an assessment of the expression of proteins associated with antioxidant and anti-inflammatory mechanisms may prove beneficial in expanding our current knowledge base. This analysis could be incorporated into future research in order to gain a deeper insight into the molecular pathways involved.

## 3. Materials and Methods 

### 3.1. Plant Material and EO Isolation 

*Citrus sinensis* (L.) Osbeck was collected from the Kenitra region (34° 15′ 39.64″ N 6° 34′ 48.72″). The botanical characterization was carried out by researchers from the Department of Biology, USMBA University, under voucher specimen BLMUP524. EO isolation was performed through hydrodistillation using a Clevenger-type device (VWR, Radnor, PA, USA) as described by [32] with some modifications. The extracted CS-EO was recuperated and kept at a temperature of 4 °C pending experimentation.

### 3.2. Chemical Profile

The chemical profile of CS-EO was subjected to analysis by gas chromatography–mass spectrometry (GC-MS) on a Shimadzu GC/MS-QP2010 system (Kyoto, Japan). The samples were vaporized and injected via split/splitless injector into a BP-X25 capillary column measuring 30 m in length and 0.25 mm in diameter. The stationary phase coating this column was non-polar, consisting of 95% dimethylpolysiloxane and 5% phenyl. The carrier gas employed was helium, with a flow rate of 3 milliliters per minute, and the temperature of the injector, ion source and interface was kept at 250 °C. The initial temperature program for the oven was set at 50 °C for one minute, after which it was increased at a rate of 10 °C per minute to reach 250 °C. This temperature was then maintained for one minute. The substances were ionized in electron impact (EI) mode at 70 electron-volts, with a mass range of 40 to 300 Dalton. The identification of molecules was achieved by comparing their retention times and mass spectra with those present in the NIST and ADMAS records. 

### 3.3. Modulation of Inflammation in LPS-Treated RAW 264.7 Cells 

#### 3.3.1. Cell Culture

The investigation entailed the cultivation of RAW 264.7 murine macrophage cells, procured from the American Type Culture Collection (ATCC, Manassas, VA, USA), in accordance with the established standards of the ATCC. Maintained at a thermostatic condition of 37 °C, the cells were cultured in Dulbecco’s Modified Eagle Medium (DMEM) supplemented with 10% fetal bovine serum (FBS), glucose (4.5 g/L), glutamine (4.5 mM), penicillin (100 units/mL) and streptomycin (and 100 μg/mL), ensuring ideal growth conditions.

#### 3.3.2. Cell Viability Assessment

Cell viability was assessed with meticulous precision using an MTT (3-(4,5-dimethylthiazol-2-yl)-2,5-diphenyltetrazolium bromide) colorimetric assay. Initially, RAW 264.7 cells were seeded at a density of 10^4^ cells/well (96-well plates), enabling a standardized cellular environment for subsequent analyses [71,72]. Afterward, different concentrations of the CS-EO and limonene were added, ranging from 25 to 200 µg/mL, both in the presence and absence of lipopolysaccharide (LPS) at a concentration of 1 μg/mL, for a duration of 24 h. Following the designated incubation period, MTT solution (10 µg/mL) was introduced into each well, and subsequent formazan crystal formation was solubilized in dimethyl sulfoxide (DMSO). Optical density (OD) was performed at 570 nm, with a background control at 630 nm, utilizing an ELISA microplate reader. 

### 3.4. Determination of Inflammatory Mediator 

The quantification of nitric oxide (NO) levels was undertaken via the Griess reaction assay [73]. RAW 264.7 cells, seeded at a density of 10^4^ cells per well, were subjected to varying treatments, encompassing exposure to LPS alone or in combination with CS-EO and limonene for a duration of 24 h. Supernatants from the culture milieu were subjected to the Griess reagent, facilitating the determination of intracellular nitrate levels, a stable derivative of NO, via OD at 540 nm using an ELISA microplate reader. Concurrently, intracellular levels of prostaglandin E2 (PGE2) were quantified utilizing an enzyme immunoassay (EIA) kit from R&D Systems, adhering strictly to the manufacturer’s prescribed protocols. This systematic methodology afforded comprehensive insights into the modulatory effects of the samples under investigation on NO and PGE2 production under LPS-induced inflammatory conditions, thereby significantly augmenting our understanding of the purported anti-inflammatory attributes inherent to these samples.

### 3.5. Cytotoxic Activity: Cell Viability by MTT Assay

MTT test was used to assess the inhibitory effect of CS-EO and limonene on cancer cell growth as indicated in [71,72]. Exponentially multiplying HepG2, MDA-MB-468 and HCT-15 cells were seeded into 96-well plates (10^4^ cells/well in 100 µL of medium) and allowed to adhere for 24 h. CS-EO and limonene were serially diluted with medium after being solubilized in 0.1% DMSO to reach acceptable concentrations. CS-EO and limonene were applied to cells at a variety of dosages, and they were then incubated for 72 h. Cells in the control group only received medium containing 0.1% DMSO. An amount of 200 µL of culture medium was used in place of the test compound medium, and 20 µL of MTT reagent was added before incubation at 37 °C for 4 h. The medium was taken out and 100 µL of DMSO was added before a microplate reader (Synergy HT Multi-Detection microplate reader, Bio-Tek, Winooski, VT, USA) measured absorbance at 540 nm and calculated % viability [74]. The standard was doxorubicin. The selectivity index (SI) was determined by dividing the IC_50_ value of the essential oil on normal cells by the IC_50_ value on the target cells [75]. 

### 3.6. Antioxidant Assays 

#### 3.6.1. DPPH Assay 

The antioxidant potential of CS-EO and limonene was evaluated through the assessment of the DPPH radical scavenging capacity, employing a laboratory optimized protocol with minor reform [76]. In summary, a series of CS-EO and limonene solutions were prepared spanning a range from 3.88 to 2000 µg/mL. Subsequently, 200 µL of each dilution was introduced to a solution of DPPH (0.04%) in 1.4 mL. The reaction mixture was maintained under controlled conditions (25 °C and 25 min) prior to spectrophotometric measurement at 517 nm. The standard antioxidant employed was BHT, tested at concentrations between 0.49 and 1000 µg/mL. To ensure the reproducibility of the results, each measurement was performed in triplicate. 

#### 3.6.2. FRAP Assay 

The reducing potential of CS-EO and limonene was investigated through the assessment of its capacity to reduce ferric iron Fe^3+^ to ferrous iron Fe^2+^ trough the FRAP method described by [77]. The experimental protocol was initiated with the creation of a series of diluted solutions of CS-EO (3.88 to 2000 µg/mL), which were then successively combined with phosphate buffer (0.5 mL) and 1% potassium ferricyanide (0.5 mL). Subsequently, the mixture was subjected to thermal incubation at 50 °C for 25 min, after which 10% trichloroacetic acid (0.5 mL) was added. The reaction solution was subjected to centrifugation (3500 rpm, 8 min) after which the resulting supernatant was collected and combined with hydrogen peroxide (0.75 mL) and 0.1% ferric chloride (0.75 µL). The intensity of the color developed was quantified spectrophotometrically at a wavelength of 700 nm. BHT was employed as positive control, and the results were expressed as mean standard deviation (n = 3).

#### 3.6.3. β-Carotene Test

The protective ability of CS-EO and limonene against lipid peroxidation was estimated trough the utilization of the beta-carotene bleaching method. This approach entails monitoring the discoloration of an emulsion comprising beta-carotene and linoleic acid in the presence of the test sample according to the method prescribed by [78] with some modifications. The preparation of the emulsion entailed the dissolution of 2 mg of beta-carotene in 1 mL of chloroform, with the subsequent addition of 25 µL of linoleic acid and 200 mg of Tween 80. Following the evaporation of the chloroform, 100 mL of distilled water saturated with 30% hydrogen peroxide was added, and the mixture was subjected to vigorous homogenization. The resulting emulsion was then dispensed into tubes, to which 175 µL of CS-EO, limonene or the reference antioxidant BHT (2 mg/mL) was added. The decolorization kinetics of the negative control, CS-EO, limonene and BHT were monitored by spectrophotometric measurements at a wavelength of 490 nm over a period of 100 min.

### 3.7. Dermatoprotective Effect

#### 3.7.1. Tyrosinase Assay

The experimental protocol employed in this assay is a modification of that previously described by [32]. In brief, the protocol entailed incubation of 15 µL of CS-EO and limonene with 75 µL of tyrosinase solution (33 U/mL, in 50 mM phosphate buffer, pH 6.5) for 11 min at 37 °C. Subsequently, 225 µL of the substrate L-DOPA (5 mM) was added, and the mixture was incubated for a further 20 min at 37 °C before measuring the absorbance at 510 nm. The degree of inhibition was calculated using the following formula:

Inhibition (%) = [(Abs _tyrosinase_) — (Abs _sample_)] × 100

The results were expressed as IC_50_ ± MSE, and quercetin was used as the reference compound.

#### 3.7.2. Elastase Assay 

The elastase inhibition capacity of CS-EO and limonene was evaluated in accordance with the methodology established by [79], with minor adjustments. CS-EO and limonene were dissolved in methanol at varying concentrations (0.5, 1, 2 and 3 mg/mL). For each concentration, a 50 µL sample was combined with 200 µL of elastase solution in a Tris-HCL buffer (0.2 M, Ph 8.0). Subsequently, the mixture was incubated at 25 °C for 15 min, after which 200 µL of N-succinyl-Ala-Ala-Ala-p-nitroanilide was added. Following thorough mixing, the samples were incubated at 25 °C for a further 20 min. The percentage of elastase inhibition was determined by measuring the absorbance at 410 nm, and quercetin was used as the positive control. 

### 3.8. Statistical Analysis

The statistical analysis involved conducting three separate tests (n = 3), and the resulting data were presented as mean values with accompanying SD. Data analysis was performed using GraphPad Prism 9 and XLSTAT statistics software version 2016. Mean values were compared using a one-way analysis of variance (ANOVA), followed by Tukey’s test. A *p*-value < 0.05 was supposed to be statistically significant.

## 4. Conclusions 

The present exploration provided a comprehensive analysis of the volatile components of *C. sinensis* EO, highlighting limonene as its predominant component. The antioxidant assays demonstrated that CS-EO and limonene possess moderate free radical scavenging and reducing power capabilities, reflecting their potential for oxidative stress management. Furthermore, the anti-inflammatory assays in RAW 264.7 cells supported the inhibitory effects of the oil and limonene on pro-inflammatory responses, offering insights into their anti-inflammatory mechanisms. The cytotoxic effects observed against cancer cell lines suggest that CS-EO and limonene have selective anticancer potential, although further studies are required to delineate the underlying mechanisms of action. The antityrosinase and antielastase properties suggest promising applications CS-EO and limonene in cosmeceutical formulations aimed at skin brightening and elasticity preservation given these enzymes’ pivotal functions in hyperpigmentation and skin aging processes.

## 5. Perspective

The findings provide a foundation for a range of potential avenues for further research. Firstly, in vivo studies are required to validate the properties observed in vitro. Secondly, the characterization of specific molecular targets and the evaluation of protein expression associated with biochemical mechanisms could facilitate the acquisition of more detailed mechanistic information. Thirdly, an investigation into the chemical variability of CS-EO in accordance with seasonal and climatic conditions would facilitate an evaluation of its potential for industrial and therapeutic applications. Additionally, in-depth research studies are needed to decipher the molecular pathways modulated by limonene in anti-inflammatory and anticancer activities, which may facilitate its application in targeted therapies. Assessing the stability and bioavailability of CS-EO and its major molecule limonene in formulations is also interesting to develop effective therapeutic or cosmeceutical products. Ultimately, the incorporation of CS-EO and limonene into pharmaceutical or cosmetic formulations could prove an auspicious avenue for their commercialization.

## Figures and Tables

**Table 1:** GC-MS analysis of CS-EO.

N°	Compounds	Molecular Formula	RI	Area (%)
**1**	α-pinene	C_10_H_16_	948	2.81
**2**	Thujene	C_10_H_16_	897	10.52
**3**	Myrcene	C_10_H_16_	958	5.54
**4**	Carene	C_10_H_16_	948	0.26
**5**	p-cymene	C_10_H_16_	987	1.00
**6**	D-limonene	C_10_H_16_	1018	70.15
**7**	Linalool	C_10_H_18_O	1082	2.07
**8**	γ-terpinene	C_10_H_18_O	1140	3.35
**9**	Citronellal	C_10_H_18_O	1031	0.57
**10**	Limonene oxide	C_10_H_16_O	1031	1.0
**11**	D-carvone	C_10_H_14_O	1190	0.73
Total				98%
Monoterpene hydrocarbons				92.63%
Oxygenated monoterpenes				4.37%
Others				1%

**Table 2:** IC_50_ values and selectivity indexes of CS-EO and its respective major compound limonene on cancer cell lines.

Treatments	IC_50_ Value ± SD (µg/mL)				Selectivity Index *		
	MCF-7	HCT-15	HepG2	PBMC	MCF-7	HCT-15	HepG2
CS-EO	30.93 ± 1.49	64.11 ± 5.45	15.97 ± 1.20	546.40 ± 6.72	17.64 ± 0.05	8.51 ± 0.20	34.17 ± 0.21
D-limonene	5.62 ± 0.75	7.32 ± 0.86	0.55 ± 0.01	21.35 ± 1.55	3.79 ± 0.19	2.92 ± 0.34	38.82 ± 0.10
Cisplatin	3.63 ± 0.40	6.56 ± 0.13	4.23 ± 0.66	23.45 ± 2.13	6.46 ± 0.20	3.57 ± 0.29	5.53 ± 0.21

* Selectivity index = (IC_50_ of PBMC/IC_50_ of tumor cells).

**Table 3:** Antioxidant activity of CS-EO and limonene.

	IC_50_ (µg/mL)		
	DPPH	FRAP	β-Carotene
CS-EO	82.54 ± 2.11 ^a^	117.35 ± 3.67 ^c^	67.48 ± 2.05 ^a^
Limonene	93.16 ± 1.56 ^b^	81.10 ± 2.21 ^b^	105.09 ± 1.26 ^c^
BHT	77.06 ± 0.03 ^a^	61.13 ± 0.03 ^a^	75.24 ± 0.09 ^b^

Records with the same letter in the same test indicate a non-significant difference by Tukey’s multiple range test (*p* < 0.05).

**Table 4:** Dermatoprotective effect of CS-EO and limonene.

	Tyrosinase	Elastase
	IC_50_ (µg/mL)	
CS-EO	65.72 ± 1.92 ^a^	102 ± 2.16 ^b^
Limonene	86.07 ± 1.53 ^b^	78.34 ± 1.15 ^a^
Quercetin	111.03 ± 0.1 ^c^	124.22 ± 0.07 ^c^

Records with the same letter in the same test indicate a non-significant difference by Tukey’s multiple range test (*p* < 0.05).

## Data Availability

The original contributions presented in this study are included in the article. Further inquiries can be directed to the corresponding author.
